# Heart rate variability analysis in toxic leukoencephalopathy-induced malignant catatonia: A case report

**DOI:** 10.1097/MD.0000000000035371

**Published:** 2023-11-03

**Authors:** Bahadar S. Srichawla, Vincent Kipkorir, Lawrence Hayward

**Affiliations:** a Department of Neurology, University of Massachusetts Chan Medical School, MA, USA; b Department of Medicine, University of Nairobi, University Way, Nairobi, Kenya.

**Keywords:** catatonia, cocaine-induced leukoencephalopathy, delayed post-hypoxic leukoencephalopathy, heart rate variability, malignant catatonia, toxic leukoencephalopathy

## Abstract

**Rationale:**

Toxic leukoencephalopathy, a condition resulting from exposure to toxic substances, can lead to malignant catatonia, a severe motor dysfunction with symptoms such as muscle rigidity and high-spiking fever, hypertensive urgency, and tachycardia. This case study investigates the relationship between toxic leukoencephalopathy-induced malignant catatonia and heart rate variability (HRV), a marker of autonomic nervous system function.

**Patient Concerns:**

A 51-year-old male presented to the emergency department with acute onset of progressively worsening mental status.

**Diagnoses:**

The patient was diagnosed with cocaine-induced toxic leukoencephalopathy causing malignant catatonia.

**Interventions:**

A 5-day escalating treatment regimen was instituted for the management of malignant catatonia until resolution. Daily HRV parameters in the temporal and frequency domain, geometric data, and cardiac entropy were recorded using *HRVAnalysis* v.1.2 (ANS Lab Tools). The HRV analysis was correlated with pharmacologic management, the Bush-Francis catatonia rating scale, and hemodynamic parameters, including blood pressure, heart rate, and temperature.

**Outcomes:**

The results showed a correlation between the severity and frequency of malignant catatonic episodes and the patient autonomic dysfunction. Improvement in malignant catatonia with pharmacological management was associated with an improved HRV, including elevated rMSSD, SDNN, cardiac entropy, and pNN50%.

**Lessons:**

Malignant catatonia is associated with decreased HRV, and its management is associated with an increase. This suggests a link between malignant catatonia and autonomic dysfunction, highlighting the potential benefits of treating malignant catatonia to improve autonomic function and reduce cardiovascular risk.

## 1. Introduction

Toxic leukoencephalopathy is a condition that results in damage to the white matter tracts of the brain due to exposure to toxic substances. The symptoms can vary depending on the substance that caused the condition and the amount of exposure but often include problems with movement, speech, and cognitive function. Some common causes of toxic leukoencephalopathy include exposure to lead, carbon monoxide, heroin, and certain solvents and pesticides.^[1]^ Malignant catatonia is a serious and life-threatening form of catatonia, encompassing a state of motor dysfunction. Malignant catatonia symptoms can include muscle rigidity, immobility, mutism, hypertensive urgency, tachycardia, and fever. The most common cause of malignant catatonia is a severe psychiatric illness, such as schizophrenia or bipolar disorder, but has also been observed in toxic leukoencephalopathy.^[2]^ Heart rate variability (HRV) is the variation in time between heartbeats, which is known to be influenced by the activity of the sympathetic and parasympathetic nervous systems. Low HRV has been associated with numerous neurological conditions including traumatic brain injury, subarachnoid hemorrhage, and intracerebral hemorrhage.^[3,4]^ To date, limited data are available on the association and effect of malignant catatonia on HRV.^[5]^ In this case study, we hypothesize that diffuse cerebral white matter disease adversely affects HRV and may be associated with increased severity and frequency of malignant cationic episodes as well as overall morbidity.

## 2. Materials & methods

We present the clinical characteristics, imaging data, management, HRV analysis, Bush-Francis catatonia rating scale (BFCRS), and results of a single patient with malignant catatonia due to cocaine-induced toxic leukoencephalopathy. Electrocardiogram (ECG) data was directly exported from PHILIPS Intellivue MX40 telemetry (Koninklijke Philips N.V., Netherlands) to Philips Patient Information Center (PIC iX). The raw ECG data was then extracted from the day of malignant catatonia diagnosis to the resolution of episodes with pharmacological treatment. 2-minute recordings were taken during the same time of the day at 1500 hours. It was ensured that the patient was awake and alert during this time for all recorded days and that the patient was not in a catatonic state. All artifactual traces (e.g., ectopic beats, movement, etc) were manually removed. The ECG rhythm was then reviewed by a board-certified cardiologist to ensure that there was no arrhythmia. A total of 5 days of recording was analyzed. HRV analysis was completed using *HRVAnalysis v1.2* (ANS Lab Tools).^[6]^ The time domain analysis was performed evaluating normal-to-normal (NN) intervals, the standard deviation between NN intervals (SDNN), the percentage of interval differences between adjacent R-R intervals >50 ms (pNN50%), and the root mean square of successive differences (rMSSD) between normal heartbeats. Fourier transforms were utilized for frequency domain analysis including total power (P_tot_), ultra-low frequencies [Hz] (ULF), very low frequencies (VLF), low frequencies (LF), and high frequencies (HF). In addition to analyzing the power in each frequency band, the ratio between LF and HF powers (LF/HF ratio) is reported and is often used as an index of sympathovagal balance. An increased LF/HF ratio indicates a shift toward sympathetic dominance, while a decreased ratio indicates a shift toward parasympathetic dominance. Entropy is a measure of the uncertainty or randomness of a signal, and in the context of HRV, it is used to analyze the complexity of the heart rhythm. High Shanon entropy (SE) indicates a more complex and variable heart rate pattern, which is generally associated with a healthier autonomic nervous system (ANS) function. In contrast, low Shanon entropy signifies a more regular and less variable heart rate pattern, which may be indicative of reduced adaptability to ANS or pathological conditions. Shanon entropy and its derivatives are included. The HRV analysis was then correlated with the BFCRS administered by a board-certified neurologist, and hemodynamic monitoring data that included daily mean systolic blood pressure (SBP_m_), mean heart rate (HR_m_), maximum temperature (T_max_), maximum systolic blood pressure (SBP_max_), maximum heart rate (HR_max_) and pharmacological management plan. Fever was defined as a temperature ≥100.2°F. Consent from the patient health care proxy was obtained for the publication of this manuscript.

## 3. Results

**Figure 1.**
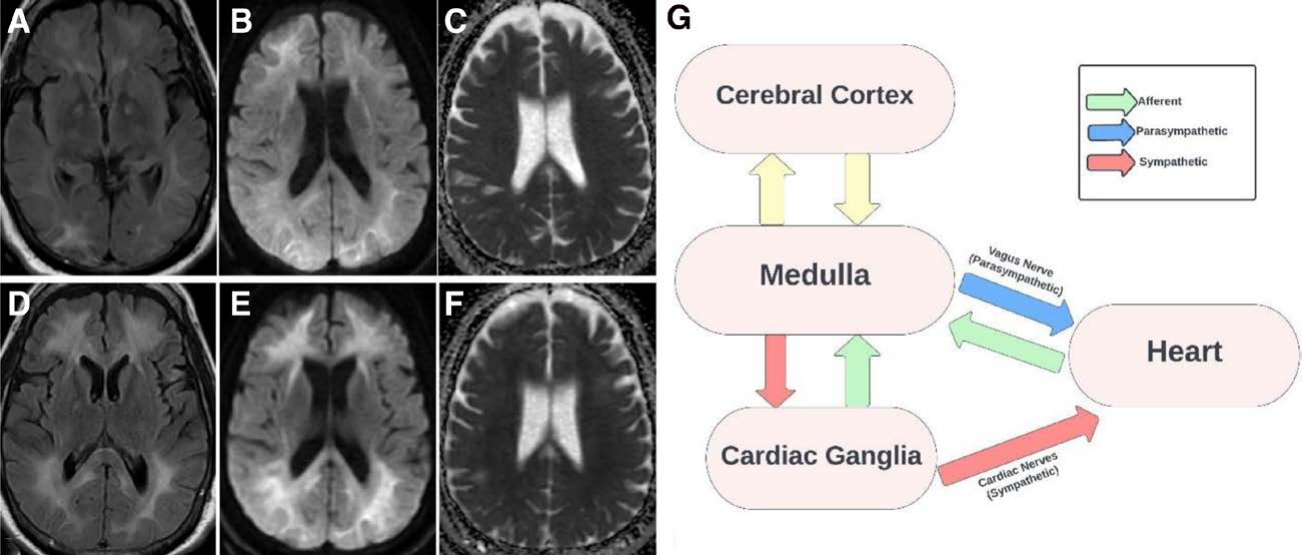
(A) MRI T2/FLAIR (B) diffusion weighted imaging (DWI) sequence and (C) apparent diffusion coefficient (ADC) on initial presentation demonstrating diffuse bilateral cortical and subcortical lesions to the white matter tracts. (D–F) Interval MRI demonstrating worsening hyperintensities in previously identified areas consistent with evolution white matter tract lesions. (G) Brain-heart communication pathways hypothesized to constitute autonomic nervous system homeostasis and heart rate variability. MRI = magnetic resonance imaging.

**Figure 2.**
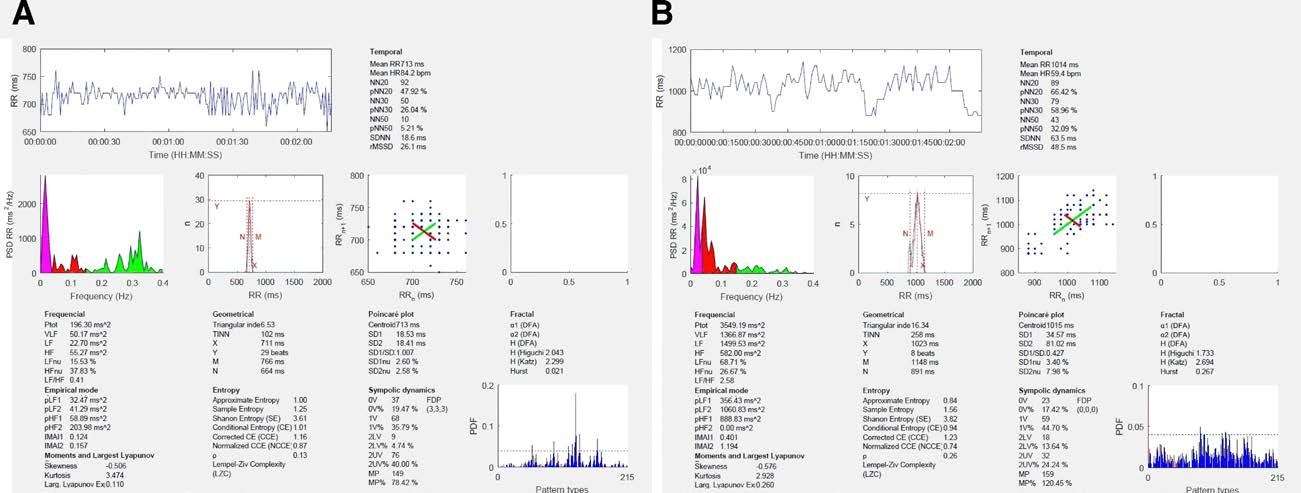
(A) HRV parameters including temporal, frequencial, entropy and geometric data on day 1 compared to (B) day 5 of treatment.

A 51-year-old male presented to the emergency department with acute onset of progressively worsening mental status. The patient had a history of drug abuse, including alcohol and intranasal cocaine. He had no relevant family medical history. On the initial examination, the patient was awake, alert, with nonsensical speech, and spontaneously moving all 4 extremities. No focal neurological deficit was observed. On the second day, the patient examination worsened, and he was globally mute, unable to respond to any commands, and began to develop rigidity in all 4 extremities. An MRI of the brain revealed diffuse hyperintense signal abnormality of T2 in cerebral white matter, with diffusion restriction on diffusion-weighted imaging and an apparent diffusion coefficient consistent with toxic leukoencephalopathy (Fig. 1A–C). An electroencephalogram (EEG) showed runs of 0.5 to 1 Hz generalized periodic discharges lasting up to 60 seconds with triphasic morphology (triphasic waves), and continuous bilateral hemispheric polymorphic slowing. The patient was started on 1000 mg of intravenous methylprednisolone for 5 days. Additionally, supplementation was initiated including oral vitamins C, E, and CoQ10. The patient clinical exam began to worsen during the second week of hospitalization, including high spiking fevers reaching 103.0°F, systolic blood pressure at 190 mm Hg, profuse hyperhidrosis, rigidity, and shivering consistent with malignant catatonia. These episodes would occur up to 5 times a day and last up to an hour. The patient required aggressive pharmacologic treatment for the management of symptoms, including lorazepam, clonidine, metoprolol, baclofen, and amantadine (Table 1). The initial rMSSD was 26.1 ms, and pNN50% was 5.21%. After 5 days of increasing therapy, the patient showed a significant improvement in the reduction of catatonic episodes and had an rMSSD of 48.5 ms and pNN50% 32.09% (Fig. 2). A repeat MRI of the brain was completed 4 weeks after the initial presentation showing interval worsening of the previous T2 cerebral white matter hyperintensities with associated diffusion restriction (Fig. 1D–F). Due to the non-improvement of symptoms, the patient was discharged to a long-term care facility. At 3 months of follow-up, the patient remained globally mute, however, all episodes of malignant catatonia had resolved.

**Table 1:** Daily heart rate variability and hemodynamic data during medication changes for the management of malignant catatonia.

Day	Medication Regimen	BFCRS	HR_max_	HR_m_	SBP_max_	SBP_m_	T_max_	rMSSD	pNN50%	SDNN	LF: HF	SE
1	Clonidine 0.1 mg TID Ativan 1 mg TID Amantadine 100 mg BID	22	154 bpm	116 bpm	186 mm Hg	162 mm Hg	102.2 F	26.1 ms	5.21%	18.6 ms	0.41	3.61
2	Clonidine 0.2 mg TID Ativan 1 mg TID Amantadine 100 mg BID	19	130 bpm	92 bpm	154 mm Hg	139 mm Hg	100.8 F	24.9 ms	4.33%	23.5 ms	3.48	3.81
3	Clonidine 0.2 mg TID Ativan 1 mg TID Amantadine 100 mg BID Baclofen 10 mg daily	19	97 bpm	75 bpm	158 mm Hg	135 mm Hg	100.2 F	27.7 ms	6.09%	30.9 ms	0.98	2.64
4	Clonidine 0.2 mg TID Ativan 1 mg TID Amantadine 400 mg daily Baclofen 5 mg TID	18	94 bpm	69 bpm	140 mm Hg	130 mm Hg	100.0 F	35.9 ms	12.65 %	48.7 ms	0.59	3.80
5	Clonidine 0.2 mg TID Ativan 1 mg TID Amantadine 400 mg daily Baclofen 10 mg TID	17	79 bpm	61 bpm	130 mm Hg	110 mm Hg	98.8 F	48.5 ms	32.09%	63.5 ms	2.58	3.82

BFCRS = Bush-Francis catatonia rating scale, LF/HF = low-frequency/high-frequency ratio, pNN50% = percentage of interval differences of adjacent R-R intervals >50 ms, rMSSD = root-mean-square of successive differences, SBP = systolic blood pressure, SDNN = standard deviation between NN intervals, SE = Shanon entropy.

## 4. Discussion

The findings of this case study provide insight into the relationship between toxic leukoencephalopathy-induced malignant catatonia and heart rate variability (HRV). The observed changes in HRV parameters and their association with the clinical course of malignant catatonia suggest a potential link between cerebral white matter disease and autonomic dysfunction (Fig. 1G). The patient presented in this study had a history of substance abuse and demonstrated diffuse cerebral white matter disease consistent with toxic leukoencephalopathy. The development of malignant catatonia in this patient emphasizes the potential of toxic leukoencephalopathy to trigger such a severe neurological condition.

The analysis of HRV parameters revealed a correlation between the daily severity and frequency of catatonic episodes and the patient autonomic dysfunction. Initial measurements of rMSSD, SE, SDNN, and pNN50% demonstrated reduced HRV, directly associated with the patient elevated blood pressure, heart rate, BFCRS, and hyperthermia. With the escalation of pharmacological management, and as the patient malignant catatonia improved, there was a notable increase in the values of rMSSD, SDNN, SE, and pNN50%, indicating a potential improvement in autonomic function. This observation supports our hypothesis that diffuse acute cerebral white matter injury adversely affects HRV and may be associated with increased severity and frequency of malignant catatonic episodes, as well as overall morbidity. Chronic cerebral small vessel disease has also been associated with a reduced HRV.^[7]^ Interestingly, the LF/HF ratio increased with the improvement of malignant catatonia, indicating a decrease in vagal tone. However, the precision of the LF/HF ratio in determining the vagal tone is debated.^[8]^

Despite improvements in catatonic episodes with pharmacological management, the patient’s brain MRI showed worsening of cerebral white matter hyperintensities and diffusion restriction, indicating the progression of toxic leukoencephalopathy. Electroconvulsive therapy is a potential management strategy in malignant catatonia; however, it can be contraindicated in leukoencephalopathy and therefore was not offered in our case.^[9,10]^ Given that this is a single-case analysis, the results may not be generalizable to all cases of malignant catatonia, and additional studies with larger sample sizes are required to confirm the observed associations between HRV and malignant. Future directions include large-scale noninvasive continuous hemodynamic monitoring and analysis of HRV in patients with an acute brain injury, ideally leveraging big data tools such as machine learning to assist in neuro-prognostication and early interventions.

## 5. Conclusions

This case study highlights an association between toxic leukoencephalopathy, malignant catatonia, and autonomic dysfunction, as evidenced by changes in HRV parameters. Our results suggest that treatment of malignant catatonia can be associated with an improvement in heart rate variability, measured by rMSSD, SDNN, SE, and pNN50%. This finding is significant as heart rate variability is a marker of autonomic nervous system function and has been linked to increased morbidity and mortality in various medical conditions. This case suggests that treatment of malignant catatonia potentially could improve autonomic nervous system function and reduce cardiovascular risk in malignant catatonia. More research is needed to determine the underlying mechanisms and long-term effects of the treatment of malignant catatonia on heart rate variability.

## Author contributions

**Conceptualization:** Bahadar S. Srichawla.

**Data curation:** Bahadar S. Srichawla.

**Formal analysis:** Bahadar S. Srichawla.

**Funding acquisition:** Bahadar S. Srichawla.

**Investigation:** Bahadar S. Srichawla.

**Methodology:** Bahadar S. Srichawla.

**Project administration:** Bahadar S. Srichawla.

**Resources:** Bahadar S. Srichawla.

**Software:** Bahadar S. Srichawla, Vincent Kipkorir.

**Supervision:** Bahadar S. Srichawla, Lawrence Hayward.

**Validation:** Bahadar S. Srichawla.

**Visualization:** Bahadar S. Srichawla.

**Writing – original draft:** Bahadar S. Srichawla.

**Writing – review & editing:** Bahadar S. Srichawla, Vincent Kipkorir, Lawrence Hayward.
